# TGFbeta and miRNA regulation in familial and sporadic breast cancer

**DOI:** 10.18632/oncotarget.14899

**Published:** 2017-01-30

**Authors:** Katia Danza, Simona De Summa, Rosamaria Pinto, Brunella Pilato, Orazio Palumbo, Massimo Carella, Ondina Popescu, Maria Digennaro, Rosanna Lacalamita, Stefania Tommasi

**Affiliations:** ^1^ IRCCS ‘Giovanni Paolo II’, Molecular Genetics Laboratory, Bari 70124, Italy; ^2^ IRCCS ‘Casa Sollievo della Sofferenza’, Medical Genetics Unit, San Giovanni Rotondo 71013, Italy; ^3^ IRCCS ‘Giovanni Paolo II’, Anatomopathology Unit, Bari 70124, Italy; ^4^ IRCCS ‘Giovanni Paolo II’, Experimental Medical Oncology Unit, Bari 70124, Italy

**Keywords:** breast cancer, TGF-β pathway, BRCA1, ATM, miRNA

## Abstract

The term ‘BRCAness’ was introduced to identify sporadic malignant tumors sharing characteristics similar to those germline BRCA-related. Among all mechanisms attributable to BRCA1 expression silencing, a major role has been assigned to microRNAs. MicroRNAs role in familial and sporadic breast cancer has been explored but few data are available about microRNAs involvement in homologous recombination repair control in these breast cancer subgroups. Our aim was to seek microRNAs associated to pathways underlying DNA repair dysfunction in breast cancer according to a family history of the disease. Affymetrix GeneChip microRNA Arrays were used to perform microRNA expression analysis in familial and sporadic breast cancer. Pathway enrichment analysis and microRNA target prediction was carried out using DIANA miRPath v.3 web-based computational tool and miRWalk v.2 database. We analyzed an external gene expression dataset (E-GEOD-49481), including both familial and sporadic breast cancers. For microRNA validation, an independent set of 19 familial and 10 sporadic breast cancers was used. Microarray analysis identified a signature of 28 deregulated miRNAs. For our validation analyses by real time PCR, we focused on miR-92a-1*, miR-1184 and miR-943 because associated to TGF-β signalling pathway, ATM and BRCA1 genes expression. Our results highlighted alterations in miR-92a-1*, miR-1184 and miR-943 expression levels suggesting their involvement in repair of DNA double-strand breaks through TGF-beta pathway control.

## INTRODUCTION

Breast cancer (BC) is a complex disease characterized by high level of heterogeneity, different clinicopathological features, prognoses and sensitivity to treatment. BC occurs both in sporadic and hereditary forms although these last represent only 5-10% of all BCs [1]. Among all the variables conferring highest women's risk of developing of BC, mutations in BRCA1 and BRCA2 genes are the best factors described. BRCA1 and BRCA2 proteins play a pivotal role in leading high-fidelity repair of double-strand DNA breaks. When mutated, homologous recombination repair (HRR) mechanism becomes “error prone”, affecting chromosomal stability and genomic integrity [2, 3]. Over the past decade, impressive advances in understanding BRCA1 and BRCA2 role in HRR allowed the development of targeted therapeutic approaches. Currently, germiline BRCA1/2 mutations represent one of the selection criteria adopted to enroll BC patients to clinical trials based on poly (ADP-ribose) polymerase inhibitors (PARPi) treatment. However, emerging data suggest that also tumors not bearing BRCA1/2 mutations but exhibiting BRCA-related gene defects and DNA repair dysfunctions are potentially responsive to PARPi-based therapy [4]. Recently, the term ‘BRCAness’ has been introduced to identify sporadic tumors sharing clinicopathological and molecular characteristics similar to those associated to BRCA1/2 germline mutations [5]. Either somatic BRCA mutations and BRCA1 gene promoter hypermethylation have been described in sporadic BC as alternative mechanisms of BRCA inactivation and BRCA-like behavior [6, 7]. Among all mechanisms attributable to BRCA1 expression silencing, a major role has been assigned to microRNAs (miRNAs) [8]. MiRNAs are a class of small non-coding RNA of 20-27 nucleotides that act as negative regulators of gene expression at post transcriptional level [9]. Our previous study reported a miRNA expression pattern able to better classify familial BC highlighting a driver role for estrogen-receptor [10]. Moreover, we showed that BRCA-related and sporadic TNBCs shared a same miRNA cluster, suggesting a similar epigenetic regulation in these tumor subgroups.

**Figure 1:**
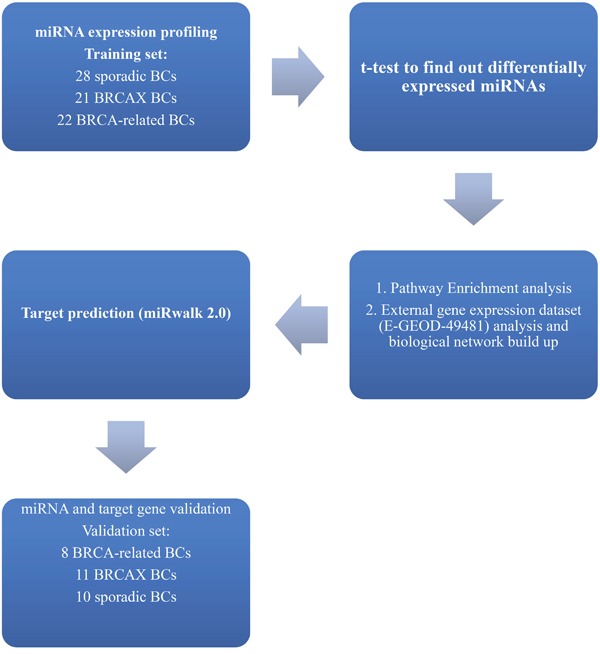
Workflow describing the study design

The role of miRNAs in familial and sporadic breast tumors has been well explored [11–14] but few data are available about miRNAs involvement in HRR control in these BC subgroups. Taking into account the relevance of the identification of sporadic tumors with BRCA-like behavior useful to PARPi treatment [15], our aim was to seek miRNAs associated to pathways underlying DNA repair dysfunction in BC according to a family history of the disease. The study design has been described in Figure 1. Our data highlighted the involvement of miR-92a-1*, miR-943 and miR-1184 deregulation in the impairment of DNA repair through TGF-β pathway control in sporadic BC.

## RESULTS

### MiRNA expression profiling in familial and sporadic breast cancer

**Figure 2:**
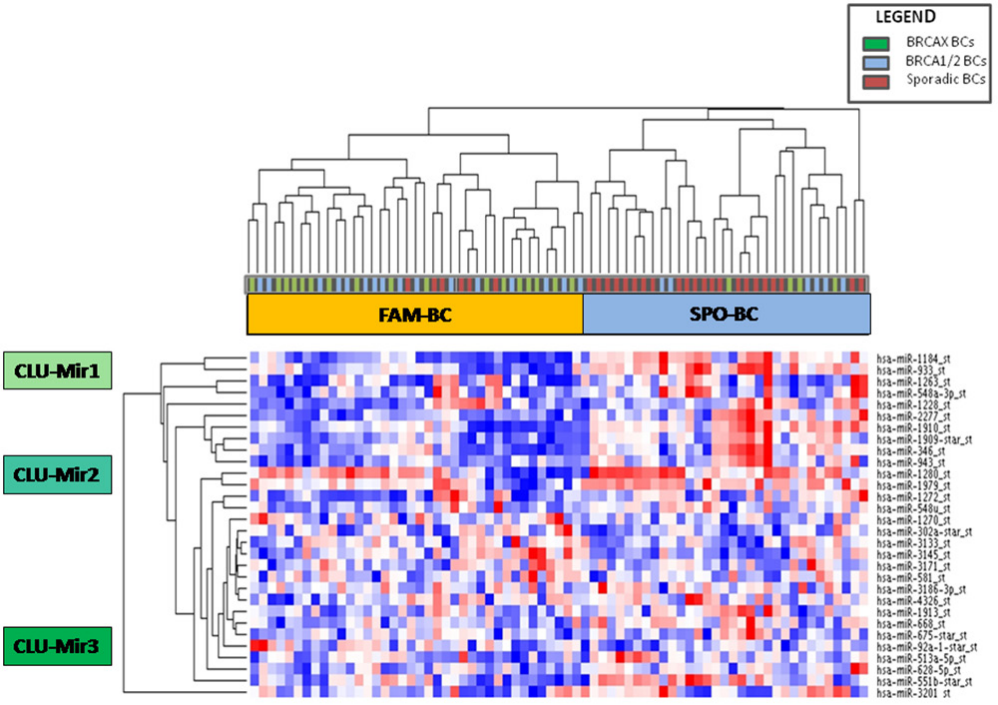
Unsupervised hierarchical clustering showing 28 differentially expressed miRNAs between familial (green and blue squares) and sporadic breast cancer cases (red squares) which cluster in two groups called FAM-BC and SPO-BC Moreover, the three miRNA clusters CLU-Mir1, CLU-Mir2 and CLU-Mir3 are indicated. (Pink indicates overexpressed miRNAs; blue indicates underexpressed miRNAs).

MiRNA expression profile was performed on a training set of 43 familial (22 BRCA1/2-related and 21 BRCAX) and 28 sporadic BC by microarray analyses. We selected miRNAs annotated as “hsa” in order to exclusively analyze the differential expression of human genes. The selected hsa-miRNAs (n=1100) were analyzed by the *t*-test. Twenty-eight miRNAs were found to be significantly differentially expressed (p<0.01) between BC with or without a positive family history of the disease (Table 1). Moreover, unsupervised hierarchical clustering (Figure 2) showed the presence of 2 sample clusters FAM-BC and SPO-BC including 76,7% and 78,5% of familial and sporadic BC, respectively.

**Table 1:** Mean intensity level of fluorescence of miRNAs differentially expressed between sporadic and familial breast tumors with a statistical significance p<0.01

miRNA	Sporadic BCs (mean±SD)	Familial BCs (mean±SD)	p-value
miR-1184	5.07±1.73	3.53±1.37	0.0003
miR-1228	4.87±1.52	3.81±1.15	0.0031
miR-1263	4.5±1.5	3.47±0.89	0.0023
miR-1270	2.43±0.21	2.64±0.42	0.0084
miR-1272	3.43±0.76	2.87±0.58	0.0019
miR-1909-star	4.19±1.4	3.35±0.91	0.0078
miR-1910	6.22±1.87	5.00±1.72	0.0075
miR-1913	3.25±0.51	2.86±0.36	0.0013
miR-2277	3.73±1.27	2.89±0.58	0.0026
miR-302a-star	2.31±0.17	2.49±0.31	0.0033
miR-3133	2.33±0.18	2.5±0.26	0.0016
miR-3145	2.27±0.2	2.44±0.29	0.0051
miR-3171	2.27±0.19	2.43±0.31	0.0079
miR-3186-3p	2.6±0.21	2.46±0.2	0.0094
miR-3201	4.03±1.25	5.16±2.09	0.0059
miR-346	4.50±1.52	3.61±0.99	0.0090
miR-4326	2.52±0.24	2.34±0.18	0.0017
miR-513a-5p	3.04±0.67	2.62±0.34	0.0043
miR-548a-3p	5.22±1.8	3.91±1.18	0.0016
miR-548u	3.03±0.62	2.65±0.48	0.0085
miR-551b-star	4.96±1.03	3.91±0.97	0.0001
miR-581	2.22±0.14	2.36±0.26	0.0066
miR-628-5p	3.47±0.87	2.9±0.52	0.0036
miR-668	2.79±0.34	2.55±0.28	0.0039
miR-675-star	2.84±0.54	2.52±0.2	0.0058
miR-92a-1-star	2.44±0.23	2.74±0.5	0.0014
miR-933	6.77±1.82	5.39±1.78	0.0027
miR-943	3.64±1.27	2.86±0.55	0.0044

Among all deregulated miRNAs, we identified 20 miRNAs up-regulated and 8 miRNAs down-regulated in sporadic BC grouped in three classes: CLU-Mir1, CLU-Mir2 and CLU-Mir3.

### Pathway enrichment analysis and targets prediction

Diana mirPath v.3 web-based computational tool was used to investigate whether the co-expression of the 28 miRNAs found deregulated between familial and sporadic BCs could affect signaling pathways associated to DNA repair.

KEGG pathway enrichment analysis (p<0.05) revealed that the set of differentially expressed miRNAs are associated with 62 different pathways among which we focused on the TGF-β signalling pathway. It has been demonstrated that TGF-β is involved in DNA damage response by down- regulating *BRCA1* and *ATM*, thus both genes expression was also investigated.

**Figure 3:**
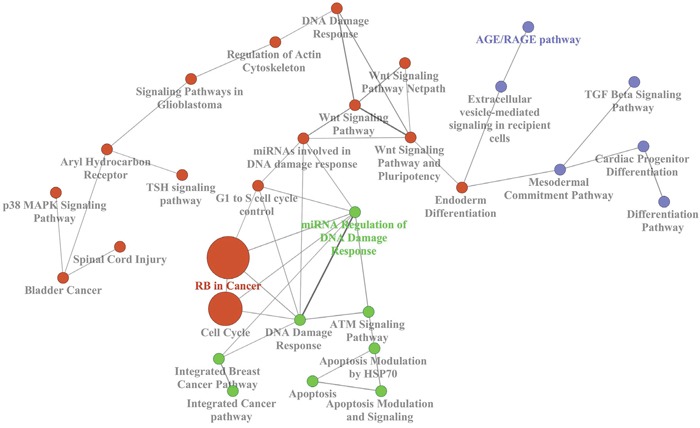
Biological network of the most represented KEGG terms of the deregulated genes in the external dataset E-GEOD-49481

Furthermore, we analyzed an external gene expression dataset (E-GEOD-49481), including both familial and sporadic BCs, to verify enriched pathways on gene level. Deregulated genes were used to build a biological network including the most significant pathways (Figure 3). Interestingly, the involvement of “TGF-β signalling pathway” and “DNA damage response” was found by the network of gene ontology. Therefore, we focused on deregulated miRNAs which were predicted to target the above mentioned three genes belonging to those pathways: *TGFB1*, *ATM* and *BRCA1*.

MiRwalk v.2 prediction tool was used in order to identify the putative targets of miRNAs correlated with TGF-β pathway or *BRCA1* and *ATM* genes. Among all miRNAs belonging to our deregulated dataset, we chose one for each miRNA-cluster (CLU-Mir1, CLU-Mir2, CLU-Mir3) highlighted by our previous microarray analyses (Figure 2), taking into account the results of pathway enrichment analysis. In particular, we focused on miR-1184 (CLU-Mir1), miR-943 (CLU-Mir2) and miR-92a-1* (CLU-Mir3). Computational analysis revealed *TGFB1* as a predictive target of miR-92a-1*, while *ATM* and *BRCA1* as potential targets of miR-1184 and miR-943.

### Validation of miRNAs

**Figure 4:**
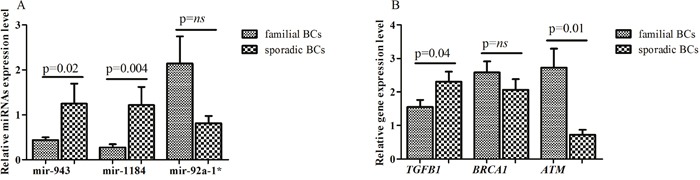
**(A)** Mean expression level of miR-943, miR-1184 and miR-92a-1* and **(B)** mean levels of TGFB1, BRCA1 and ATM genes.

Validation analysis, performed by real-time PCR on an independent set of 19 familial (8 BRCA1/2-related and 11 BRCAX) and 10 sporadic BCs, showed a lower expression of miR-92a-1* in sporadic BCs compared to familial breast tumors (0.81 ± 0.16 vs 2.14 ± 0.6; p=0.12) and a statistically significant higher mean expression levels of miR-943 and miR-1184 in BCs without a positive family history compared with familial breast tumors subgroup (1.24 ± 0.44 vs 0.43 ± 0.06; p=0.02 and 1.21 ± 0.4 vs 0.27 ± 0.07; p=0.004, respectively) (Figure 4A). Considering the median expression level as cutoff, the percentage of both miR-943 and miR-1184 overexpression was significantly higher in sporadic BC patients compared to those with a positive family history of the disease (miR-943: 90% vs 31,6%; p= 0.005 and miR-1184: 90% vs 31,6%; p= 0.005). No difference in percentage of miR-92a-1* expression was found between the two BC subgroups.

### TGFB1, ATM and BRCA1 transcripts levels

*TGFB1*, *ATM* and *BRCA1* genes expression was performed by real time PCR. The mean expression level of *TGFB1* gene was higher in sporadic compared with familial BC (2.30 ± 0.29 vs 1.55 ± 0.21; p= 0.04), whereas the mean level of ATM transcript was lower in sporadic BC compared to breast tumors with a family history (0.72 ± 0.15 vs 2.72 ± 0.56; p= 0.001). Although it did not reach a statistical significance, a lower *BRCA1* gene expression was found in sporadic compared to familial BCs (2.06 ± 0.31 vs 2.57 ± 0.33; p= 0.33) (Figure 4B). Furthermore, sporadic BCs showed a higher percentage of *TGFB1* over-expression compared to familial breast tumors (60 vs 42.1 %; p=0.44). On the contrary, higher frequency of *ATM* and *BRCA1* gene over-expression was observed in familial compared to sporadic BC groups (63.15 vs 30%; p=0.12 and 58 vs 30%; p=0.44, respectively). Although not statistically significant, a negative correlation was found between miR-92a-1*/*TGFB1* (Pearson r= -0.28; p= 0.13), miR-1184/*ATM* (Pearson r= -0.2; p= 0.29), miR-1184/*BRCA1* (Pearson r=-0.2; p= 0.28), miR-943/*ATM* (Pearson r= -0.15; p= 0.41) and miR-943/*BRCA1* (Pearson r= -0.16; p= 0.37).

## DISCUSSION

Mutations in *BRCA1* and *BRCA2* genes are the best described factors which contribute to increase women's risk for developing BC. The risk is principally associated to the role of BRCA1 and BRCA2 in repairing DNA double-strands breaks. Currently, malignant tumors harboring BRCA1/2 mutations are those selected for PARPi treatment but emerging evidence suggests that also tumors with defects in other components of DNA damage pathways may benefit from these drugs. The term ‘BRCAness’ was introduced to describe the sporadic tumors exhibiting histomorphological features and immunophenotypic profile similar to those bearing *BRCA1/2* germline mutations [16]. Among all mechanisms directly attributable to BRCAness, *BRCA1* promoter hypermethylation and somatic *BRCA1/2* mutations have been proposed [16–18]. Recently, microRNAs emerged as pivotal players in regulating the activity and level of components in the DNA damage response pathways [19]. Major efforts concern the possible identification of sporadic BCs with a molecular behavior similar to BRCA-related patients treatable with PARPi [15]. Thus, the aim of this study was to investigate whether signaling pathways related to DNA damage repair mechanisms could be affected by miRNAs deregulation in breast tumors according to a family history. Previously, we showed that a subset of sporadic BCs, triple-negative or not, had a similar miRNA expression profile to those of BRCA-related cases [10], suggesting that the BRCAness phenotype could not be simply inferred from histopathological features. The role of miRNAs in both familial and sporadic BC has been previously investigated. In particular, Bastos EP and coworkers identified a miRNA signature capable to discriminate familial from sporadic non-BRCA1/2 breast carcinoma in patients younger than 35 years [11]. Moreover, other reports revealed the function of miRNAs in detecting BRCA genetic or epigenetic alterations in hereditary and sporadic breast tumors or finally, in discerning sporadic and BRCA1 associated basal-like BC [12–14]. However, this is the first study investigating whether miRNAs that are capable to stratified sporadic and familial BC could affect pathways associated to DNA damage repair mechanisms in order to highlight novel markers for sporadic BC therapy. Our microarray analysis identified a signature of 28 deregulated miRNAs, almost all of which up-regulated in sporadic breast tumors. Interestingly, in line with the literature, cluster analysis on the whole training set showed that most of familial and sporadic BCs clustered independently. Because of the deregulation of a set of miRNA could influence cellular functions by affecting multiple pathways [20], we performed a pathway enrichment analysis in order to explore whether our deregulated miRNAs could be associated to DNA damage response signals. Out of the 62 cellular pathways found, we specifically focused on that involving TGF-β. Recently it has been demonstrated that TGF-β induces a genomic instability by regulating DNA repair. An opposite effect of TGFB1/Smad3 and BRCA1 on repair DNA mechanism and genomic integrity has been reported [21]. TGF-β is a multifunctional regulator of diverse cellular processes including cell growth, apoptosis, angiogenesis, differentiation and migration. Both tumor-suppressing and tumor-promoting functions have been described for TGF-β in BC [22] but, more recently, a key role in regulating DNA repair damage has been also observed [23–25]. Interestingly, when the analysis of an external gene expression dataset including familial and sporadic BCs was performed, we found the involvement of both “TGF-β signalling pathway” and “DNA damage response” supporting our data. Among all miRNAs associated to TGF-β signalling control, we chose miR-92a-1*, miR-1184 and miR-943 belonging to the three miRNA-clusters independently (Mir-CLU1, Mir-CLU2 and Mir-CLU3), as highlighted by our analyses. In our validation analyses, although not statistically significant, a lower miR-92a-1* levels and statistically significant higher miR-1184 and miR-943 mean expression levels were found in sporadic breast tumors with respect to those with a positive family history of the disease. In addition, when we explored *TGFB1* expression, higher level of this gene was detected in sporadic BC. Recently, it has been demonstrated that TGF-β can induce ‘BRCAness’ phenotype and sensitivity to PARPi in BC cells without *BRCA* genes mutations by suppressing the expression of genes related to DNA repair damage mechanisms. In particular, TGF-β seems to down-regulate the expression of mutS homolog 2 (*MSH2*) [26], ataxia telangiectasia (*ATM*) [27] and *BRCA1* genes through a miRNA-mediated mechanism, leading to an impaired DNA repair efficiency [23]. As expected, in our series, lower levels of both *BRCA1* and *ATM* genes were observed in sporadic breast tumors in which higher expression of *TGFB1* was observed. Besides *ATM* and *BRCA1* regulation by TGFB1 via miRNA mechanism, we assumed that both genes could be directly controlled by miR-1184 and miR-943 as highlighted by a negative relationship between their expression levels, although they did not reach a statistical significance. In the last years, the attention has been drawn to molecular aberrations underlying DNA repair damage dysfunction in order to better select patients treatable with PARPi. A recent study showed the possibility of considering the overexpression of PARP1 and miR-151-5p as biomarkers useful to correctly treat sporadic breast cancers with PARPi [28].

In conclusion, miRNA-mediated DNA repair damage through TGF-β signaling was investigated in BC with respect to family history of the disease. Using two independent BC patient sets, our results showed alterations in miR-92a-1*, miR-1184 and miR-943 expression levels in familial and sporadic BC, suggesting an involvement in repair of DNA double-strand breaks through TGF-beta pathway control. These data need further investigation in a larger cohort but highlighted the relevance of miR-92a-1*, miR-1184 and miR-943 to stratify sporadic BC who may benefit from personalized therapy.

## MATERIALS AND METHODS

### Patients

BC tissues used as training set were obtained from 71 female patients enrolled at the IRCCS “Giovanni Paolo II” of Bari and stratified into 43 familial (22 BRCA1/2-related and 21 BRCAX) and 28 sporadic tumors. DNA from peripheral blood was screened for all BRCA1 and BRCA2 gene mutations and BC that showed clinical characteristics as previously reported [29] were identified as those with a positive family history of the disease and followed in our Genetic Counseling Program. The study was approved by the Ethics Committee of the same Institute as a satellite project of the protocol approved with n. 56/CE of 16/05/2011. All patients signed an informed consent for authorizing the research and all data have been processed with respect for privacy and anonymity. Validation analysis were performed on an independent set consisting of 19 familial and 10 sporadic BC. Moreover, the normal tissues counterpart of a representative number of both familial and sporadic BC samples were utilized as control in real time analysis.

### MiRNA expression profiling

Ten μm-thick formalin-fixed, paraffin-embedded sections were used to extract total RNA, including microRNAs, using the RNeasy® FFPE Kit (QIAGEN) according to the manufacturer's protocol. 500 ng of RNA of each sample was labelled using the 3DNA Array Detection FlashTagTM RNA Labeling Kit according to the manufacturer's instructions, and analyzed with the GeneChip miRNA v. 1.0 Array (Affymetrix). This contains 46,228 probes comprising 7,815 probe sets, and covers 71 organisms including 848 human miRNAs derived from the Sanger miRBase and miRNA database v11 (April 15, 2008, http://microrna.sanger.ac.uk). Firstly, poly (A) tailing was carried out at 37°C for 15 min in a volume of 15 ml reaction mix, which contained 1X Reaction Buffer, 1.5 ml MgCl_2_ (25 mM), 1 ml ATP Mix diluted 1:500, and 1 ml PAP enzyme. Secondly, Flash Tag Ligation was performed at room temperature for 30 min by adding 4 ml of 5X Flash Tag Ligation Mix Biotin and 2 ml T4 DNA Ligase into the 15 ml of reaction mix. To stop the reaction, 2.5 ml of Stop Solution was added. Each sample was hybridized on the array, washed, and stained with the Affymetrix Fluidics Station 450. They were then scanned with the Affymetrix GeneChip Scanner 3000 7G using the Command Console software (Affymetrix). Microarray dataset has been deposited at ArrayExpress database (https://www.ebi.ac.uk/arrayexpress/) under the accession number E-MTAB-2705 (https://www.ebi.ac.uk/arrayexpress/experiments/E-MTAB-2705/). Unsupervised average-linkage hierarchical clustering using Pearson's correlation was performed through ‘Hierarchical clustering’ module and heat map was created with ‘Hierarchical clustering image’ module of Gene Pattern suite [30].

### Array data processing and statistical analysis

Raw data were normalized through the Robust Multi-array Average (RMA) method to remove systematic variations. Briefly, RMA corrects raw data for background using a formula which is based on a normal distribution and uses a linear model to estimate values on a log-scale. RMA normalization was performed using the “affy” package of the Bioconductor suite (http://www.bioconductor.org/) for the R statistical language (http://cran.r-project.org/). The default settings were used. Normalized values were statistically analyzed with MeV software v.4.8.1 (Dana-Farber Cancer Institute, Boston, MA, USA). Differentially-expressed miRNAs were detected through the t-test, and data were considered statistically significant when p<0.01.

### Pathway enrichment analysis

DIANA miRPath v.3 pathway enrichment analysis (http://www.microrna.gr/miRPathv3) [31] was used to investigated pathways related to differentially expressed miRNAs between familial and sporadic BC. DIANA miRPath is a web-based computational tool able to detect the combinatorial effect of multiple miRNAs in pathways. The software performs an enrichment analysis of multiple miRNA targets correlating each set of microRNA target genes to KEGG pathways. All pathways showing *P*-values <0.05, were considered significantly enriched between groups under comparison.

### Gene expression analysis from a public dataset and biological network construction

In order to study the gene expression pattern, pre-processed data from the dataset E-GEOD-49481 were downloaded from ArrayExpress (https://www.ebi.ac.uk/arrayexpress/). The dataset included 33 BRCA1-mutated, 22 BRCA2-mutated, 70 BRCAX and 128 sporadic breast cancer samples. Normalised values were statistically analysed with MeV software v.4.8.1 (Dana-Farber Cancer Institute, Boston, MA, USA). Differentially expressed genes (DEGs) were detected through t-test, with data considered statistically significant when p<0.01.

To build the biological network coming from DEG list, we used CLUEGO v2.2.3 [32], a Cytoscape plugin. In detail, Kegg and Wiki Path terms were included for the construction of the network.

### Quantitative miRNAs RT-PCR analysis

MiRNA and mRNA expression analyses were performed on an independent set of breast tumors stratified into 19 familial (8 BRCA1/2-related and 11 BRCAX) and 10 sporadic BCs.

Total RNA was extracted from formalin-fixed, paraffin-embedded breast cancers, as described above. Briefly, for detection of miR-92a-1*, miR-1184 and miR-943 expression levels, 10 ng of total RNA were reverse transcribed using the TaqMan^®^ MicroRNA Reverse Transcription Kit and miRNA specific primers according to the manufacturer's protocol (Applied Biosystems). Real Time PCR analysis was performed on the ABI Prism 7000 Sequence Detection System (Applied Biosystems) using 3 μl of RT products in a reaction mixture containing TaqMan miRNA assay and the TaqMan Universal PCR Master Mix, according to the manufacturer's instructions (Applied Biosystems). All PCR reactions were performed in triplicate including no-template controls. Relative quantities of each miRNA were calculated using the ΔΔCt method after normalization with endogenous reference RNU 48.

### Quantitative RT-PCR analysis of *TGFB1*, *BRCA1* and *ATM* genes

For gene expression analysis, 400 ng of total RNA were reverse transcribed in 20 μl using the High Capacity cDNA Reverse Transcription Kit, according to the manufacturer's protocol (Applied Biosystem). Quantitative real-time PCR was performed on the ABI Prism 7000 Sequence Detection System (Applied Biosystems) in accordance to the manufacturer's instructions (Applied Biosystems),. The ID assays used were the following: human TGFB1 (Hs00998133_m1), human BRCA1 (Hs01556193_m1), human ATM (Hs01112307_m1). RN18S1 (Hs03928985_g1) was used as the endogenous reference. Relative expression was calculated using the comparative Ct method. All PCRs were performed in triplicate including no-template controls.

### Computational and statistical analysis

The MiRWalk v2.0 database [33] was used to identify predicted miRNAs targets. Computational analysis revealed *TGFB1* (RNA hybrid) as a predictive target of miR-92a-1*, *ATM* (miRWalk, miRanda, RNA hybrid, Targetscan, RNA22) and *BRCA1* (miRMap, RNA22, RNA hybrid, miRWalk, Targetscan) as potential targets of miR-1184. Furthermore, *ATM* (RNA hybrid, miRWalk, Microt4, MiRanda, RNA22, Targetscan, MiRMap) and *BRCA1* (Microt4, RNA22, RNA hybrid, miRWalk) were also highlighted as potential targets of miR-943.

Data analysis was performed using the GraphPad Prism statistics software package (GraphPad Prism 5.0). Statistical significance was determined using the Student's t test and the two-tailed Fisher's exact test. Expression values are reported as mean±SEM. Pearson's correlation coefficient, r, was used to describe the association between miRNAs and their targets. Values of *P*<0.05 were considered statistically significant.
